# Dual diagnosis of achondroplasia and mandibulofacial dysostosis with microcephaly

**DOI:** 10.1186/s12920-024-01999-0

**Published:** 2024-09-06

**Authors:** Ekaterina Lyulcheva-Bennett, Christopher Kershaw, Eleanor Baker, Stuart Gillies, Emma McCarthy, Jenny Higgs, Natalie Canham, Dawn Hennigan, Chris Parks, Daimark Bennett

**Affiliations:** 1 Liverpool Centre for Genomic Medicine, Liverpool Women’s NHS Foundation Trust, Liverpool, L8 7SS UK; 2 Faculty of Health and Life Sciences, University of Liverpool, Liverpool, L69 7ZB UK; 3 North West Genomic Laboratory Hub, St Mary’s Hospital, Oxford Road, Manchester, M13 9WL UK; 4 North West Genomic Laboratory Hub, Liverpool Women’s Hospital, Liverpool, L8 7SS UK; 5 Department of Neurosurgery, Alder Hey Children’s NHS Foundation Trust, Liverpool, L14 5AB UK; 6 Faculty of Biology, Medicine and Health, University of Manchester, Michael Smith Building, Manchester, M13 9PT UK

**Keywords:** Achondroplasia, Mandibulofacial dysostosis with microcephaly, Dual molecular diagnosis, Whole genome sequencing, Deep phenotyping, Blended phenotype, Genetic counselling

## Abstract

**Background:**

Achondroplasia and mandibulofacial dysostosis with microcephaly (MFDM) are rare monogenic, dominant disorders, caused by gain-of-function *fibroblast growth factor receptor 3* (*FGFR3*) gene variants and loss-of-function *elongation factor Tu GTP binding domain-containing 2* (*EFTUD2*) gene variants, respectively. The coexistence of two distinct Mendelian disorders in a single individual is uncommon and challenges the traditional paradigm of a single genetic disorder explaining a patient’s symptoms, opening new avenues for diagnosis and management.

**Case Presentation:**

We present a case of a female patient initially diagnosed with achondroplasia due to a maternally inherited pathogenic *FGFR3* variant. She was referred to our genetic department due to her unusually small head circumference and short stature, which were both significantly below the expected range for achondroplasia. Additional features included distinctive facial characteristics, significant speech delay, conductive hearing loss, and epilepsy. Given the complexity of her phenotype, she was recruited to the DDD (Deciphering Developmental Disorders) study and the 100,000 Genomes project for further investigation. Subsequent identification of a complex *EFTUD2* intragenic rearrangement confirmed an additional diagnosis of mandibulofacial dysostosis with microcephaly (MFDM).

**Conclusion:**

This report presents the first case of a dual molecular diagnosis of achondroplasia and mandibulofacial dysostosis with microcephaly in the same patient. This case underscores the complexity of genetic diagnoses and the potential for coexistence of multiple genetic syndromes in a single patient. This case expands our understanding of the molecular basis of dual Mendelian disorders and highlights the importance of considering the possibility of dual molecular diagnoses in patients with phenotypic features that are not fully accounted for by their primary diagnosis.

**Supplementary Information:**

The online version contains supplementary material available at 10.1186/s12920-024-01999-0.

## Introduction

The Occam’s razor principle [1], which suggests that the simplest explanation is often the best, has traditionally guided the assumption in genetics that a single diagnosis is more likely than multiple independent ones. However, advances in Genomic Medicine and high-throughput sequencing technologies have challenged this paradigm, revealing that dual molecular diagnoses can be more common than expected [2–4]. This shift underscores the importance of deep phenotyping to identify inconsistent features and the possibility of a blended phenotype, which can guide genomic testing, clinical management, and genetic counselling.

Achondroplasia is the most prevalent cause of disproportionate short stature, with an estimated prevalence of approximately 1 in 25,000 [5]. It is characterised by rhizomelic limb shortening, macrocephaly and characteristic facial features such as frontal bossing and midface retrusion [6, 7]. Hypotonia is common in infancy, often leading to some delays in the acquisition of developmental motor milestones. Cognitive function and lifespan are typically unaffected, although craniocervical junction compression increases the risk of death in infancy. Additional complications include obstructive sleep apnoea, middle ear dysfunction leading to conductive hearing loss, kyphosis, and spinal stenosis [6–8]. Achondroplasia is caused by gain-of-function variants in *FGFR3*, which negatively impacts the growth of long bones by inhibiting chondrocyte proliferation and differentiation in the growth plate [8, 9]. In cases of diagnostic uncertainty or atypical findings, identification of a heterozygous pathogenic variant in *FGFR3* can establish the diagnosis. Achondroplasia is inherited in an autosomal dominant manner, with around 80% of cases resulting from a *de novo* pathogenic variant [6].

Mandibulofacial dysostosis with microcephaly (MFDM) is classified among the facial dysostoses, a group of rare and heterogeneous genetic congenital malformation syndromes that result from disrupted development of the first and second pharyngeal arches [10–12]. MFDM is characterised by microcephaly, distinctive craniofacial features, and variable intellectual disability [13, 14]. The condition is caused by variants in the *EFTUD2* gene [15], which encodes a component of the spliceosome, a multiprotein complex involved in the splicing of pre-mRNA. EFTUD2 haploinsufficiency leading to aberrant splicing is thought to be the underlying mechanism for MFMD. Previously reported pathogenic variants include missense, nonsense, frameshift, and splice site variants, as well as whole or partial deletions of EFTUD2 [15–19]. Individuals with MFDM typically present with malar and mandibular hypoplasia, microcephaly and learning difficulties [20]. Other clinical features include external ear malformations with conductive hearing loss, epilepsy and variable short stature in some patients [14, 20]. Skeletal abnormalities in MFDM may include abnormalities of the middle ear ossicles, thumb anomalies such as triphalangeal thumbs, and vertebral anomalies. Other skeletal anomalies in MFDM that affect the craniofacial complex are cleft palate, choanal atresia, zygomatic arch cleft. Imaging studies can demonstrate these skeletal abnormalities as well as the characteristic craniofacial features [14, 20]. MFDM is a rare condition, with 126 cases reported to date in the medical literature [14]. MFDM follows an autosomal dominant pattern of inheritance. Most cases of MFDM are caused by *de novo* gene variants. However, a small number of cases have been reported in which an affected individual inherits the variant from an affected parent [14, 20, 21].

## Case presentation

### Patient and clinical evaluation

**Fig. 1:**
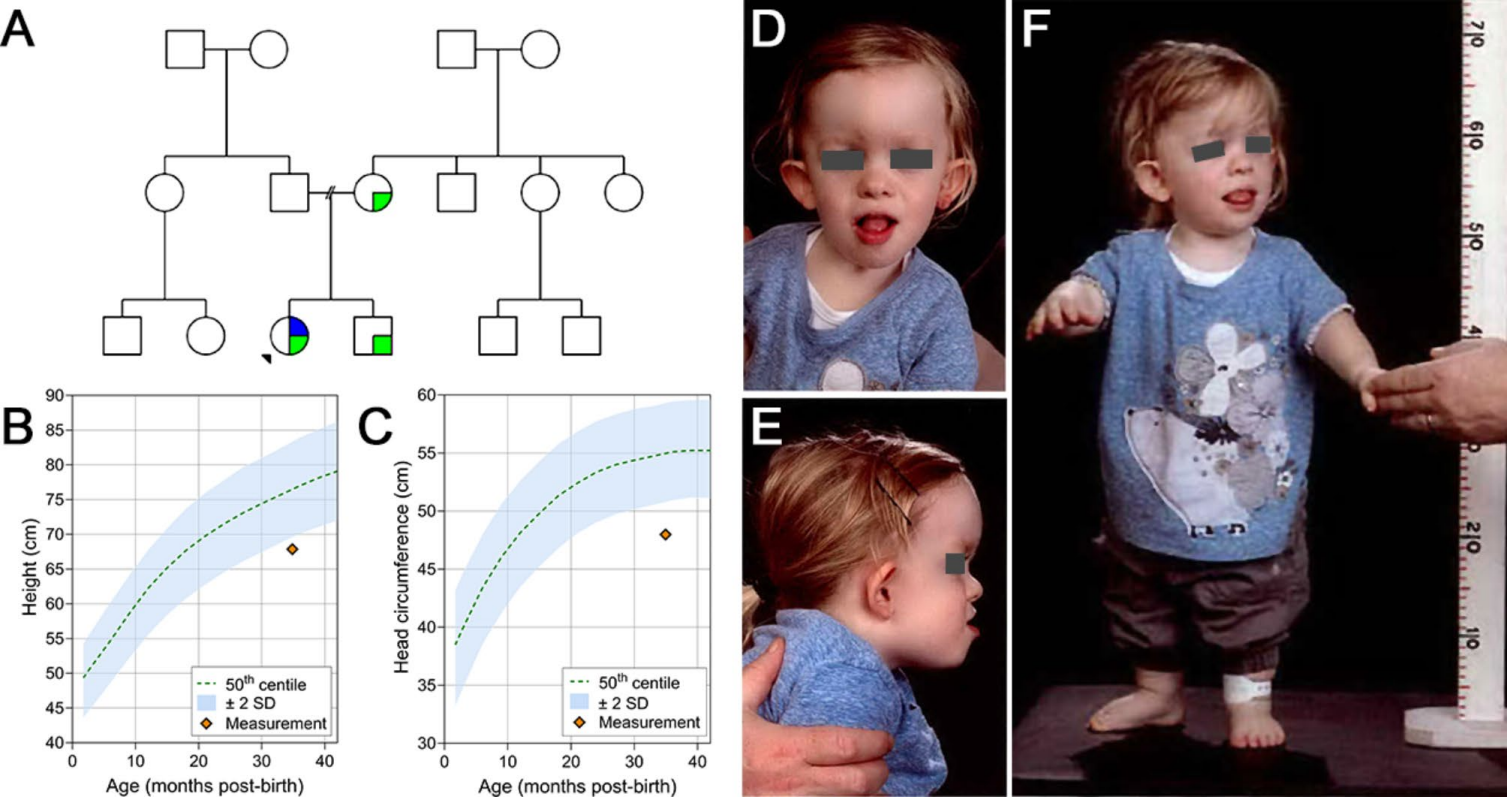
Pedigree and clinical presentation **A**, Family pedigree. Black arrow shows the proband. Green quadrant indicates the patient or family members affected with achondroplasia, caused by a variant in the *FGFR3* gene. Blue quadrant indicates patient with additional features that are not fully explained by familial *FGFR3* variant. **B**, Achondroplasia height growth chart. **C**, Achondroplasia head circumference growth curve. **D-E**, Distinctive dysmorphic facial features from front (**D**) and side (**E**), including prominent, dysplastic, low-set ears with absent antihelices; down-slanting palpebral fissures; midface hypoplasia with a prominent mandible, frontal bossing and depressed nasal bridge. **F**, Rhizomelic limb shortening, characteristic of achondroplasia, exhibited by the patient. **D-F**, Patient’s eyes have been covered to protect privacy

A 3-year-old girl with disproportionate short stature was initially diagnosed with achondroplasia due to a maternally inherited pathogenic *FGFR3* variant (*FGFR3*, NM_000142.5: c.1138G > A, p.Gly380Arg), that also affected her older brother (Fig. 1a). However, her growth rate and height were significantly below the mean for achondroplasia (Fig. 1b, c). She was also noted to be normocephalic, which is not in keeping with achondroplasia and indeed represents significant microcephaly on achondroplasia growth charts (Fig. 1c).

The patient exhibited significant speech and language delay, learning difficulties, conductive hearing loss, and epilepsy (onset of seizures at age 2). Distinctive features included prominent, dysplastic, low-set ears with absent antihelices giving them a protruding impression; down-slanting palpebral fissures; midface and malar hypoplasia with a prominent mandible, frontal bossing and depressed nasal bridge (Fig. 1d; Table 1).

**Table 1:** Patient’s blended phenotype consists of features of both achondroplasia and MFDM

Clinical Features	achondroplasia	MFDM	Our Patient
Short stature	Yes	Yes	Yes ++
Macrocephaly	Yes	No	No
Microcephaly	No	Yes	Yes (relative)^*^
Midface hypoplasia	Yes	No	Yes
Malar hypoplasia Dysplastic ears	No No	Yes Yes	Yes Yes
Speech delay	Yes	Yes	Yes
Learning Difficulties	No	Yes	Yes
Conductive hearing loss	Yes	Yes	Yes
Seizures	No	Yes	Yes

*Relative microcephaly, defined as having an occipitofrontal circumference beyond 2 standard deviations below the mean for age in patients with achondroplasia (according to achondroplasia growth charts)

Imaging showed features consistent with achondroplasia. MRI of the brain showed small posterior fossa and small foramen magnum causing effacement of the cerebrospinal fluid (CSF) spaces at the craniocervical junction. There was also some bossing of the forehead and expansion of the extra-axial CSF spaces and mild ventricular dilation. The torcula was also low with a vertical straight sinus. MRI of the spine showed exaggerated thoracic kyphosis and thoracolumbar kyphosis and a narrow, but not critically stenotic, spinal canal. The kyphotic deformities have improved with time. To date, no surgical intervention has been needed for these issues.

### Genetic screening and results

Given the complexity of the patient’s phenotype and limited relevant family history, the patient was recruited to both the Deciphering Developmental Disorders (DDD) study [22] and the 100,000 Genomes project [23, 24]. Genomic DNA was extracted from peripheral blood samples using the Chemagen DNA Extraction kit, following the manufacturer’s instructions. Trio whole-genome sequencing (WGS) was performed on DNA from the whole blood using the Illumina platform (HiSeq 2500, 150 bp paired-end reads). The data were aligned to the reference genome. Initial analysis confirmed the presence of a maternally inherited *FGFR3* variant (*FGFR3*, c.1138G > A), but was otherwise uninformative.

However, subsequent re-analysis of the WGS data indicated the presence of the complex rearrangement within the *EFTUD2* gene. To confirm the WGS findings, PCR primers were designed to amplify across the gene, resulting in a 6.7 kb product for the wild type allele in control gDNA. The patient’s DNA sample showed two distinct PCR products sizes, the 6.7 kb product indicating a wild type allele, and a shorter 756 bp allele, indicating a deletion event (supplementary information).

**Fig. 2:**
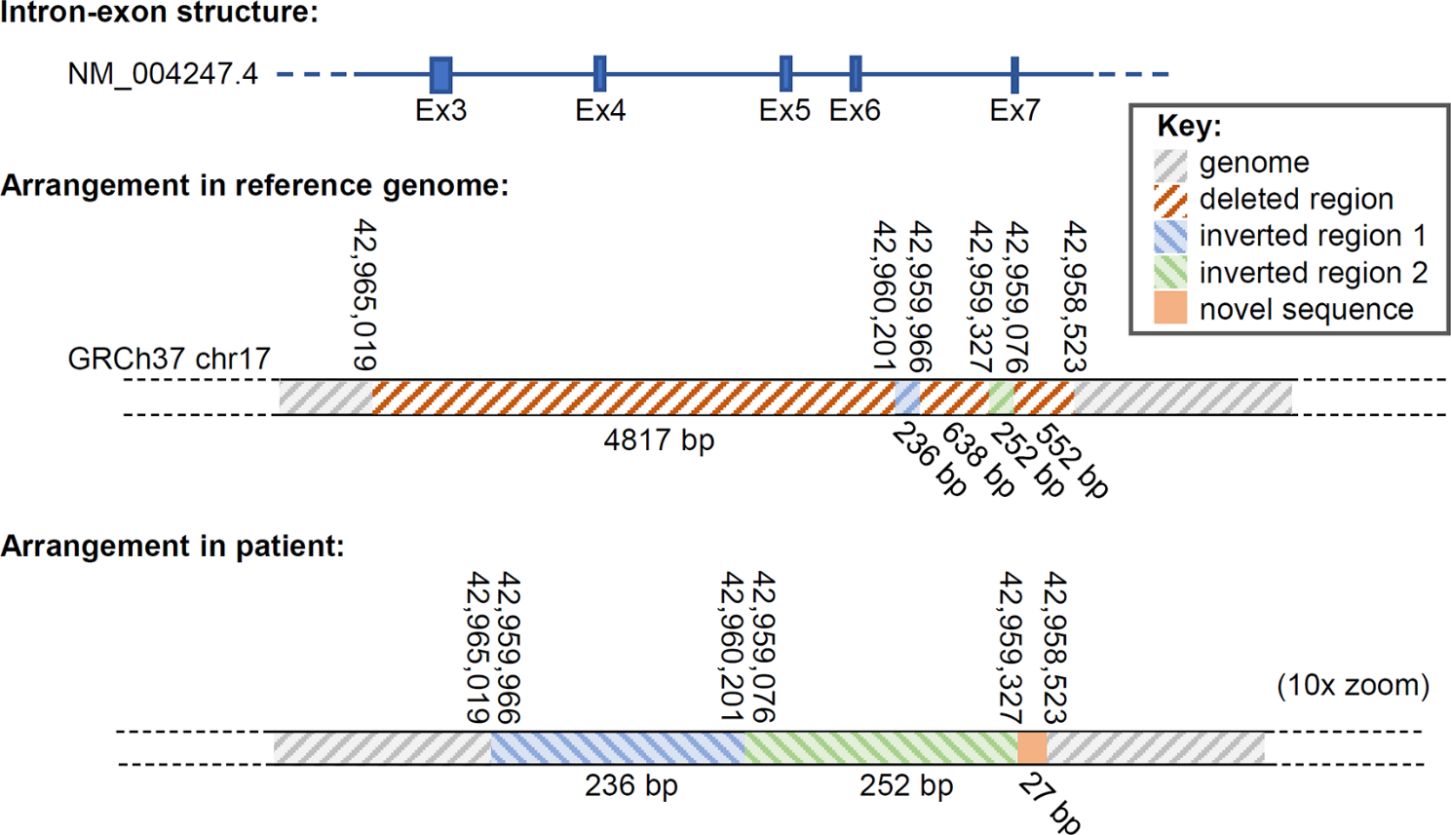
*EFTUD2* intragenic rearrangement in the patient. Top, genomic organisation of the affected part of the *EFTUD2* gene, showing exons (ex) 3–7, interspersed with non-coding intronic sequence. Middle, annotation of this region of *EFTUD2* locus in the reference genome, showing parts of the gene that harbour deletions, inversions or novel sequence in the patient. Colour coding is shown in the key. Bottom, annotation of the patient’s *EFTUD2* gene in this region, shown at 10x zoom. Nucleotide numbers refer to positions in the reference genome

To elucidate the nature of the shorter 756 bp PCR product detected in the patient’s DNA sample, the fragment was sequenced in duplicate in both directions. Manual analysis of the Sanger sequencing data revealed a complex intragenic rearrangement comprised of two inverted regions, three deleted regions (including deletion of *EFTUD2* exons 3–6 and partial deletion of exon 7) and a novel sequence that yielded no nucleotide search matches using BLAST (Fig. 2 and supplementary information). This insertion-deletion (InDel) was consistently identified in eight separate sequencing attempts. Testing of parental samples showed a lack of the InDel, indicating this variant occurred *de novo* in the patient.

The patient’s additional phenotypic features were consistent with MFDM, although choanal atresia cleft palate or zygomatic arch cleft were not observed in our patient. The complex intragenic rearrangement variant was classified as pathogenic in line with the Association for Clinical Genomic Science (ACGS) best practice guidelines for variant interpretation (https://www.acgs.uk.com/quality/best-practice-guidelines/), based on being predicted to elicit nonsense-mediated decay, absence from control individuals in the gnomAD v3.1 dataset and *de novo* status in this patient. Accordingly, an additional diagnosis of MFDM was made in our patient. We conclude that this patient exhibits a blended phenotype, with features of both achondroplasia and MFDM (Table 1).

## Discussion and conclusions

In this case, we present a patient with a unique combination of achondroplasia and MFDM, conditions typically associated with variants in *FGFR3* and *EFTUD2* genes, respectively. These genes are both involved in fundamental biological processes regulating growth, albeit through different mechanisms.

*FGFR3* encodes a receptor for fibroblast growth factors, which are involved in a variety of biological processes including cell growth, morphogenesis, tissue repair, and tumour growth [9]. Gain-of-function variants result in the constitutive activation of FGFR3 in the absence of ligand binding. This activates several important downstream signalling pathways that disrupt the normal bone growth, resulting in disproportionate short stature and other clinical features of achondroplasia [8, 9]. Our patient displayed rhizomelic limb shortening, frontal bossing and midface hypoplasia, typical of constitutively activated FGFR3.

On the other hand, heterozygous variants in *EFTUD2* cause MFDM, a disorder characterised by craniofacial abnormalities, short stature, and intellectual disability [14, 20]. Our patient’s smaller than expected head circumference, dysplastic ears, seizures, and intellectual disability are consistent with MFDM. Although the exact mechanism by which *EFTUD2* variants cause these features is not fully understood, EFTUD2 haploinsufficiency is thought to be the underlying mechanism for MFMD [15, 20]. Loss of function *EFTUD2* variants are thought to disrupt normal splicing [17, 19] and thus affect the expression of multiple genes, potentially including those involved in growth and development. *EFTUD2* has been shown to be an essential gene in mice, with null mutants causing early embryonic lethality. [25]

Although both FGFR3 and EFTUD2 impact growth, they operate through distinct pathways: FGFR3 affects bone growth via chondrocyte proliferation and differentiation, while EFTUD2 influences growth more broadly through its role in gene expression. There is no known direct interaction between these genes, but it is possible that their variants could synergistically enhance short stature, a hypothesis that warrants further research.

This case underscores the complexity of genetic disorders and the interplay of different biological processes in growth and development. It also highlights the importance of comprehensive genetic testing in providing accurate diagnosis and guiding management for patients with complex phenotypes. Clinical WGS frequently utilises virtual gene panels or other variant filtration methods (e.g. using HPO-terms). If the phenotype is atypical, it is more likely that a molecular diagnosis will be missed. This emphasises the need for deep phenotyping and the recognition of blended phenotypes to ensure that all relevant gene panels are considered in the analysis.

Genetic counselling for individuals with dual molecular diagnoses and their families presents its own set of complexities. The reproductive risks and options in such cases are multifaceted and can be challenging to navigate. Preimplantation genetic diagnosis (PGD), a procedure used to help identify genetic alterations within embryos prior to implantation, may be technically challenging when two genetic disorders are involved.

In addition to the proband’s clear reproductive risks, parents of a child with a dual molecular diagnosis also face recurrence risks. Where both parents are affected, the likelihood of offspring inheriting at least one of the autosomal dominant conditions is very high (75%). In each pregnancy, there is a 25% chance that the child will be unaffected by either condition, a 25% chance that the child will be affected by both conditions, and a 50% chance the child will be affected by either one condition or the other. Even in cases where parents are unaffected, there is a risk that the relevant genetic variant is present in a portion of their reproductive cells. The incidence of germline mosaicism varies by disorder and is estimated to be around 6% in MFDM due to sequence variants [13]. This phenomenon means that even in apparent simplex cases, there is a low risk of an unexpected recurrence in subsequent pregnancies, necessitating careful genetic counselling.

The increased identification of dual molecular diagnoses represents a significant shift in the field of Genomic Medicine. It challenges the traditional paradigm of a single genetic disorder explaining a patient’s presentation and opens new possibilities for diagnosis and management. It is important to remember that in an individual with a pre-existing familial disorder, the risk of a second *de novo* pathogenic variant occurring will be similar to the risk in the general population. The coexistence of multiple genetic syndromes in a single patient is likely an under-recognised phenomenon. As our understanding of genetic disorders continues to evolve, it is crucial that we consider the possibility of dual molecular diagnoses in patients with phenotypic features that are not fully accounted for by their primary diagnosis, to provide the best possible care for patients.

## Electronic supplementary material

Below is the link to the electronic supplementary material.

Supplementary Material 1

## Data Availability

The variants described in his report have been submitted to DECIPHER (ID: 301978) and de-identified genomic datasets are available to registered members of the Genomics England Research Network. Information regarding how to join the Genomics England Research Network and apply for data access is available at the following URL: https://www.genomicsengland.co.uk/research/academic/join-gecip.

## Abbreviations

ACGS: Association for Clinical Genomic Science
BLAST: Basic Local Alignment Search Tool
BLAT: BLAST-like Alignment Tool
CSF: Cerebrospinal fluid
EFTUD2: Elongation factor Tu GTP binding domain containing 2
FGFR3: Fibroblast growth factor receptor 3
MFDM: Mandibulofacial dysostosis with microcephaly
SD: Standard deviation
WGS: Whole genome sequencing

## References

[1] Van Den Berg HA. Occam’s razor: from Ockham’s via moderna to modern data science. Sci Prog. 2018;101(3):261–72. 30025552 10.3184/003685018X15295002645082 PMC10365162

[2] Balci TB, Hartley T, Xi Y, Dyment DA, Beaulieu CL, Bernier FP, Dupuis L, Horvath GA, Mendoza-Londono R, Prasad C, et al. Debunking Occam’s razor: diagnosing multiple genetic diseases in families by whole-exome sequencing. Clin Genet. 2017;92(3):281–9. 28170084 10.1111/cge.12987

[3] Posey JE, Harel T, Liu P, Rosenfeld JA, James RA, Coban Akdemir ZH, Walkiewicz M, Bi W, Xiao R, Ding Y, et al. Resolution of Disease Phenotypes resulting from Multilocus genomic variation. N Engl J Med. 2017;376(1):21–31. 27959697 10.1056/NEJMoa1516767 PMC5335876

[4] Spedicati B, Morgan A, Pianigiani G, Musante L, Rubinato E, Santin A, Nardone GG, Faletra F, Girotto G. Challenging Occam’s Razor: Dual Molecular Diagnoses Explain Entangled Clinical Pictures. *Genes (Basel)* 2022, 13(11). 10.3390/genes13112023 PMC9690221 36360260

[5] Foreman PK, van Kessel F, van Hoorn R, van den Bosch J, Shediac R, Landis S. Birth prevalence of achondroplasia: a systematic literature review and meta-analysis. Am J Med Genet A. 2020;182(10):2297–316. 32803853 10.1002/ajmg.a.61787 PMC7540685

[6] Legare JM. Achondroplasia. In: *GeneReviews((R)).* Edited by Adam MP, Feldman J, Mirzaa GM, Pagon RA, Wallace SE, Bean LJH, Gripp KW, Amemiya A. Seattle (WA); 1993.

[7] Savarirayan R, Ireland P, Irving M, Thompson D, Alves I, Baratela WAR, Betts J, Bober MB, Boero S, Briddell J, et al. International Consensus Statement on the diagnosis, multidisciplinary management and lifelong care of individuals with achondroplasia. Nat Rev Endocrinol. 2022;18(3):173–89. 34837063 10.1038/s41574-021-00595-x

[8] Horton WA, Hall JG, Hecht JT. Achondroplasia. Lancet. 2007;370(9582):162–72. 17630040 10.1016/S0140-6736(07)61090-3

[9] L’Hote CG, Knowles MA. Cell responses to FGFR3 signalling: growth, differentiation and apoptosis. Exp Cell Res. 2005;304(2):417–31. 15748888 10.1016/j.yexcr.2004.11.012

[10] Wieczorek D. Human facial dysostoses. Clin Genet. 2013;83(6):499–510. 23565775 10.1111/cge.12123

[11] Bukowska-Olech E, Materna-Kiryluk A, Walczak-Sztulpa J, Popiel D, Badura-Stronka M, Koczyk G, Dawidziuk A, Jamsheer A. Targeted next-generation sequencing in the diagnosis of facial dysostoses. Front Genet. 2020;11:580477. 33262786 10.3389/fgene.2020.580477 PMC7686794

[12] Terrazas K, Dixon J, Trainor PA, Dixon MJ. Rare syndromes of the head and face: mandibulofacial and acrofacial dysostoses. Wiley Interdiscip Rev Dev Biol 2017, 6(3). 10.1002/wdev.263 PMC5400673 28186364

[13] Huang L, Vanstone MR, Hartley T, Osmond M, Barrowman N, Allanson J, Baker L, Dabir TA, Dipple KM, Dobyns WB, et al. Mandibulofacial Dysostosis with Microcephaly: mutation and database update. Hum Mutat. 2016;37(2):148–54. 26507355 10.1002/humu.22924 PMC5512564

[14] Lines M, Hartley T, MacDonald SK, Boycott KM. Mandibulofacial Dysostosis with Microcephaly. In: *GeneReviews((R)).* Edited by Adam MP, Feldman J, Mirzaa GM, Pagon RA, Wallace SE, Bean LJH, Gripp KW, Amemiya A. Seattle (WA); 1993. 24999515

[15] Lines MA, Huang L, Schwartzentruber J, Douglas SL, Lynch DC, Beaulieu C, Guion-Almeida ML, Zechi-Ceide RM, Gener B, Gillessen-Kaesbach G, et al. Haploinsufficiency of a spliceosomal GTPase encoded by EFTUD2 causes mandibulofacial dysostosis with microcephaly. Am J Hum Genet. 2012;90(2):369–77. 22305528 10.1016/j.ajhg.2011.12.023 PMC3276671

[16] Deml B, Reis LM, Muheisen S, Bick D, Semina EV. EFTUD2 deficiency in vertebrates: identification of a novel human mutation and generation of a zebrafish model. Birth Defects Res Clin Mol Teratol. 2015;103(7):630–40. 10.1002/bdra.23397 PMC4487781 26118977

[17] Beauchamp MC, Djedid A, Bareke E, Merkuri F, Aber R, Tam AS, Lines MA, Boycott KM, Stirling PC, Fish JL, et al. Mutation in Eftud2 causes craniofacial defects in mice via mis-splicing of Mdm2 and increased P53. Hum Mol Genet. 2021;30(9):739–57. 33601405 10.1093/hmg/ddab051 PMC8161524

[18] Lei L, Yan SY, Yang R, Chen JY, Li Y, Bu Y, Chang N, Zhou Q, Zhu X, Li CY, et al. Spliceosomal protein eftud2 mutation leads to p53-dependent apoptosis in zebrafish neural progenitors. Nucleic Acids Res. 2017;45(6):3422–36. 27899647 10.1093/nar/gkw1043 PMC5389467

[19] Thomas HB, Wood KA, Buczek WA, Gordon CT, Pingault V, Attie-Bitach T, Hentges KE, Varghese VC, Amiel J, Newman WG, et al. EFTUD2 missense variants disrupt protein function and splicing in mandibulofacial dysostosis Guion-Almeida type. Hum Mutat. 2020;41(8):1372–82. 32333448 10.1002/humu.24027

[20] Lehalle D, Gordon CT, Oufadem M, Goudefroye G, Boutaud L, Alessandri JL, Baena N, Baujat G, Baumann C, Boute-Benejean O, et al. Delineation of EFTUD2 haploinsufficiency-related phenotypes through a series of 36 patients. Hum Mutat. 2014;35(4):478–85. 24470203 10.1002/humu.22517

[21] Guion-Almeida ML, Vendramini-Pittoli S, Passos-Bueno MR, Zechi-Ceide RM. Mandibulofacial syndrome with growth and mental retardation, microcephaly, ear anomalies with skin tags, and cleft palate in a mother and her son: autosomal dominant or X-linked syndrome? Am J Med Genet A. 2009;149A(12):2762–4. 19921636 10.1002/ajmg.a.32816

[22] Firth HV, Wright CF, Study DDD. The Deciphering Developmental disorders (DDD) study. Dev Med Child Neurol. 2011;53(8):702–3. 21679367 10.1111/j.1469-8749.2011.04032.x

[23] Investigators GPP, Smedley D, Smith KR, Martin A, Thomas EA, McDonagh EM, Cipriani V, Ellingford JM, Arno G, Tucci A, et al. 100,000 genomes pilot on rare-disease diagnosis in Health Care - Preliminary Report. N Engl J Med. 2021;385(20):1868–80. 34758253 10.1056/NEJMoa2035790 PMC7613219

[24] Turnbull C, Scott RH, Thomas E, Jones L, Murugaesu N, Pretty FB, Halai D, Baple E, Craig C, Hamblin A, et al. The 100 000 genomes project: bringing whole genome sequencing to the NHS. BMJ. 2018;361:k1687. 29691228 10.1136/bmj.k1687

[25] Beauchamp MC, Djedid A, Daupin K, Clokie K, Kumar S, Majewski J, Jerome-Majewska LA. Loss of function mutation of Eftud2, the gene responsible for mandibulofacial dysostosis with microcephaly (MFDM), leads to pre-implantation arrest in mouse. PLoS ONE. 2019;14(7):e0219280. 31276534 10.1371/journal.pone.0219280 PMC6611600

