# Supplementary material for: Dual diagnosis of achondroplasia and mandibulofacial dysostosis with microcephaly
Source: BMC Med Genomics. 2024 Sep 6;17:226. doi: 10.1186/s12920-024-01999-0 (PMC11378366; doi:10.1186/s12920-024-01999-0)
Supplement: Supplementary file 1 — Supplementary Material 1 [file 12920_2024_1999_MOESM1_ESM.pdf]

Supplemental Data for:
Dual Diagnosis of Achondroplasia and Mandibulofacial Dysostosis with Microcephaly

Ekaterina Lyulcheva-Bennett[1,2,*], Christopher Kershaw[3], Eleanor Baker[3], Stuart Gillies[4], Emma McCarthy[4], Jenny Higgs[1], Natalie Canham[1], Dawn Hennigan[5], Chris Parks[5], Daimark Bennett[2,6,*]

Table of Contents, this file.

Figure S1 Snapshot of Integrative Genomics Viewer showing affected region of the EFTUD2 gene in the proband.

Figure S2 Gel electrophoresis showing PCR amplification of variant region from control and Proband gDNA.

Figure S3 Proband sequences from Sanger sequencing of the PCR product shown in Fig.S2B.

Figure S4 Sequence of genomic region of the indel in EFTUD2 in the proband.

Figure S5 Sequencing chromatograms from Sanger sequencing of the PCR product shown in Fig.S2B.

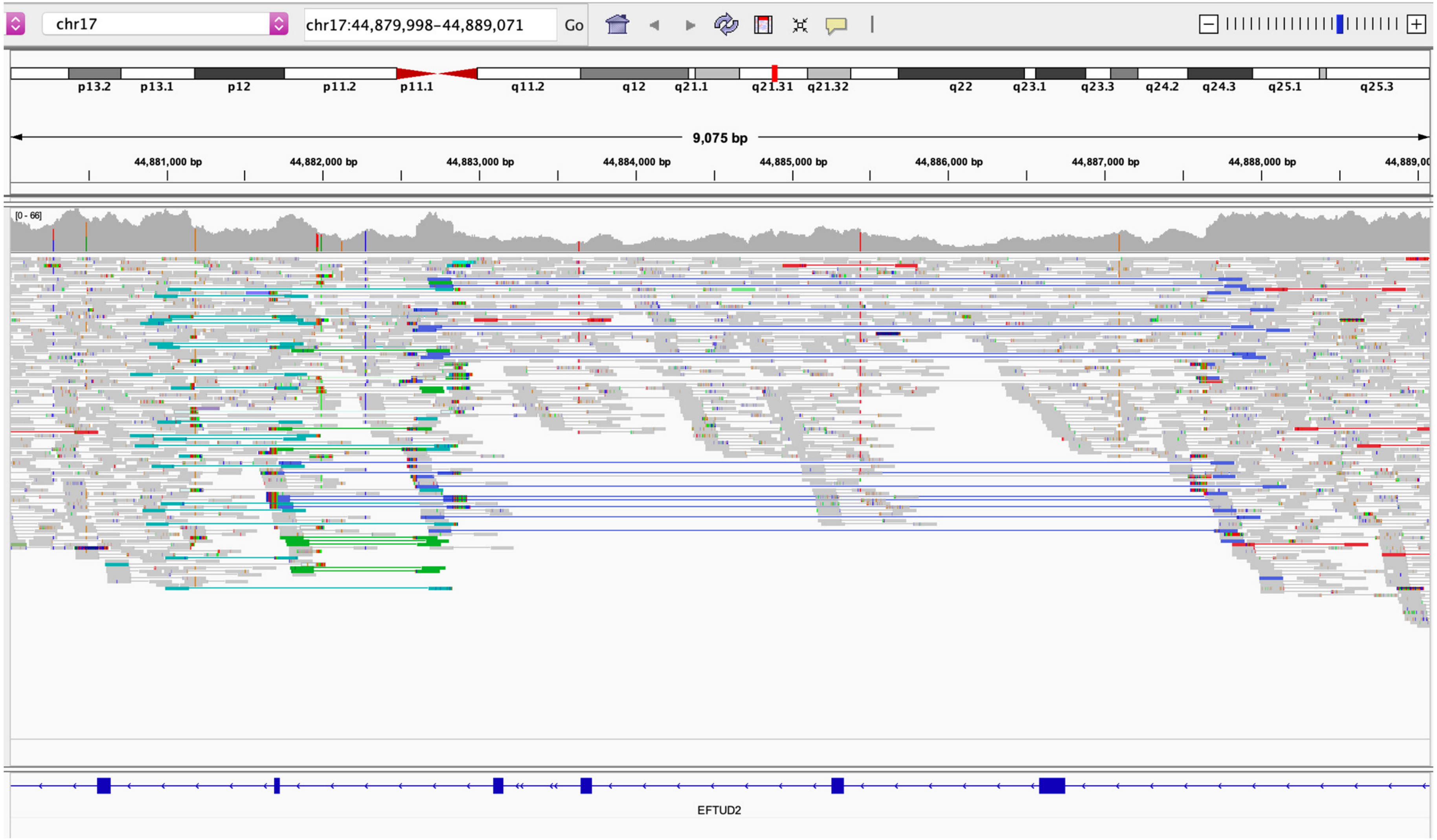

Figure S1. **Snapshot of Integrative Genomics Viewer showing affected region of the *EFTUD2* gene in the proband.**

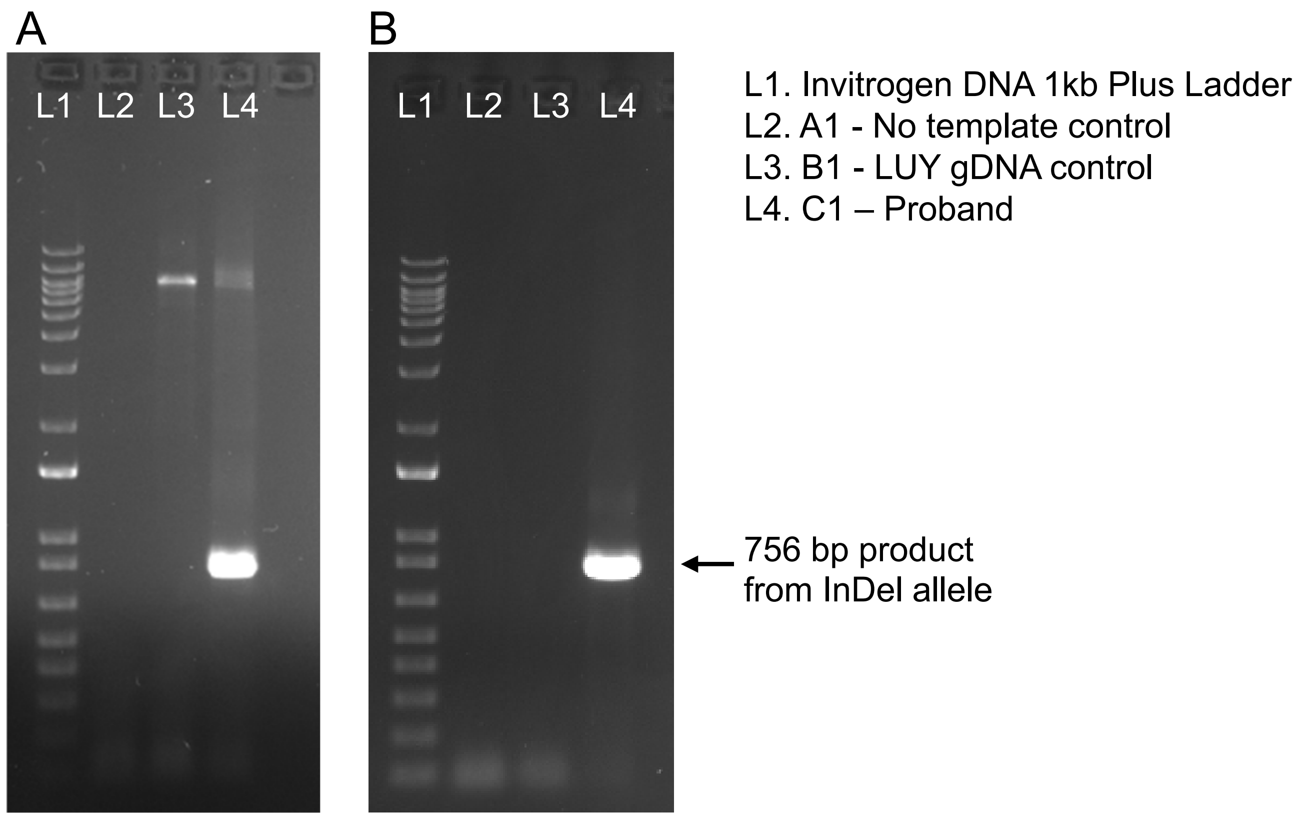

Figure S2. Gel electrophoresis showing PCR amplification of variant region from control and Proband gDNA. A 6761 bp amplification product from the unaffected allele and a 756 bp product for the InDel allele (arrow) are visible. Loading of lanes L1-L4, were as indicated. **A**, Elongation time 5 min 30 sec. **B**, Elongation time 1 min, to amplify InDel allele alone.
Primer sequences, N13 tagged to enable Sanger sequencing using standardised N13 primers were as follows: EFTUD2_F: gtagcgcgacggccagtTGGGTTAATTTGTAAGGAGGCAGA, EFTUD2_R: cagggcgcagcgatgacACAGCTAACATTGATTGCCTATCT).

A

TGGGATGGGAGGCAGATTTGAGTTATTTATTGTAAAGTGTACAATTTAATGGCTTTTAGAGTATTCACAGAGTCATGCAACTGTCATCACAATCAATTTTAGAACATT
TCCATTATCTCAGAAAAAAACTCCACACCCCTTACCCATTGTCCTCCCACCCACACCTACCCTGCCAGCCCTGGGCAATCTGCATTTATATCAAATGCACAGAATTCT
TAGTGTGGTAAAGGGAGACACATGTCAGCCTTAATGCTAAAGTGTTAAAACAATCAATCTGGCAGGGTTTGAACATTTATTGCACTGAGACTTGCAGACACAGTTGGC
CTCATTCACAGGCATTTAAATTTACCATGAAAACCAGGTACAATTGTACCATATTTATAGAGCAGGCTATTAATACTATAACAGGAAAGAATAGACTATAAAAGTCAG
TATAGCACAGCTGAAAAAAAATTAACTGTTGTTACCACCTGAGTTGAGACTGCTCTCCCACAAACGAACCATCAATCACTTTCCCTCACTACCTCCCAGCTCAAGGGT
TTCTTTGAGATTAAAGAGAGAGAGAGAGCAGGGTGTCCCCACAGCAATTACAGACAATGAGACCAGTGCCCTCCCAGCTGGAAATGCTCCTGGGTGTGTGGAGGGATT
TTTTTATTTTTACTTTTTTGGAGACAGAGTTATATGGGTATATGCAGATTTAGTTAACATAAGTATGGTTAACTAAAGGCACTTGGAATTAGTAAAGATAGATAGGCA
ATCAATGTTAGCTGTGTCATCGCCTCGCGCGCCTGTGA

B

GTTAACTAAATCTGCATATACCCATATAACTCTGTCTCCAAAAAAGTAAAAATAAAAAAATCCCTCCACACACCCAGGAGCATTTCCAGCTGGGAGGGCACTGGTCTC
ATTGTCTGTAATTGCTGTGGGGACACCCTGCTCTCTCTCTCTCTTTAATCTCAAAGAAACCCTTGAGCTGGGAGGTAGTGAGGGAAAGTGATTGATGGTTCGTTTGTG
GGAGAGCAGTCTCAACTCAGGTGGTAACAACAGTTAATTTTTTTTCAGCTGTGCTATACTGACTTTTATAGTCTATTCTTTCCTGTTATAGTATTAATAGCCTGCTCT
ATAAATATGGTACAATTGTACCTGGTTTTCATGGTAAATTTAAATGCCTGTGAATGAGGCCAACTGTGTCTGCAAGTCTCAGTGCAATAAATGTTCAAACCCTGCCAG
ATTGATTGTTTTAACACTTTAGCATTAAGGCTGACATGTGTCTCCCTTTACCACACTAAGAATTCTGTGCATTTGATATAAAT

C

TGGGATGGGAGGCAGATTTGAGTTATTTATTGTAAAGTGTACAATTTAATGGCTTTTAGAGTATTCACAGAGTCATGCAACTGTCATCACAATCAATTTTAGAACATT
TCCATTATCTCAGAAAAAAACTCCACACCCCTTACCCATTGTCCTCCCACCCACACCTACCCTGCCAGCCCTGGGCAATCTGCATTTATATCAAATGCACAGAATTCT
TAGTGTGGTAAAGGGAGACACATGTCAGCCTTAATGCTAAAGTGTTAAAACAATCAATCTGGCAGGGTTTGAACATTTATTGCACTGAGACTTGCAGACACAGTTGGC
CTCATTCACAGGCATTTAAATTTACCATGAAAACCAGGTACAATTGTACCATATTTATAGAGCAGGCTATTAATACTATAACAGGAAAGAATAGACTATAAAAGTCAG
TATAGCACAGCTGAAAAAAAATTAACTGTTGTTACCACCTGAGTTGAGACTGCTCTCCCACAAACGAACCATCAATCACTTTCCCTCACTACCTCCCAGCTCAAGGGT
TTCTTTGAGATTAAAGAGAGAGAGAGAGCAGGGTGTCCCCACAGCAATTACAGACAATGAGACCAGTGCCCTCCCAGCTGGAAATGCTCCTGGGTGTGTGGAGGGATT
TTTTTATTTTTACTTTTTTGGAGACAGAGTTATATGGGTATATGCAGATTTAGTTAACATAAGTATGGTTAACTAAAGGCACTTGGAATTAGTAAAGATAGATAGGCA
ATCAATGTTAGCTGTGTCATCGCCTCGCGCGCCTGTGA

Figure S3. Proband sequences from Sanger sequencing of the PCR product shown in Fig.S2B. A, Forward sequence, with parts in the correct orientation to the genome highlighted in yellow. **B**, Reverse complement of the unhighlighted part of (A) only, coloured as follows: green = reverse complement section 1, blue = reverse complement section 2. **C**, Proband sequence colour coded as above except, in addition, sequence of unknown origin is highlighted red.

ACCTCAAAGTTGCCACATGCTCTTGGCTCTGGGCCTTTGCATTCGCTGTTTCACCAGCCGACTAGAATGCTCTTCCCCAGGTACCTGTAGAGCTTATTCTCTGTCTGCAGATTTTTTGCTTCAGTAGCACCTCTTTTTCCTTGATTGCTTTATTACTAACACC
TGGAACAGTGCTTAGCACATATTAAGTGCTCAGTAAGTGTTTTAGGACTTAAAGGGTGATCTGGTGGCCACCTTAGATTATTTGGAAGACTTTTTAGCAGAAGTGAGAATGGCTTCGTTAATGTTGCATATGACTATGGGTTAATTTGTAAGGAGGCAGATTT
GAAGTTATTTAATTGTAAAGTGTACAATTTAATGGCTTTTAGAGTATTCACAGAGTCATGCAACTGTCATCACAATCAATTTTAGAACATTTCCATTATCTCAGAAAAAAACTCCACACCCCTTACCCATTGTCCTCCCACCCACACCTACCCTGCCAGCCCT
GGGCAATCTGCTTTCTGTCTATATAGATTCGCCTATTCTGAATATTTTCATATAAATGGAATCATATAATATGCAGCTTTTTCAGTCTGGCCTCTTTACTCAGCATCATGTTTTCAAGGCTTATCTACGCTGTAGCATGTATATACCTATAGCAAGTATAGGT
ATTTTGTTTGTTTTTGTTGCCAAATAATCTTTTGTATGGATGTACCACATTTCATTTATCCAGTCATCAGTTGATCGACATTTGGTTGTTCCCACTTTTTGGCTATTATAAATAATGCCACTATGAACATTGATTTCTAAGTTTTTGTGTGGCCATAAGTTTT
CATTTGTCTTGGGTATATACTTAGGAGTGGACTTGCTGGGTCATATGTAACTCTGTGTTGAACATTTTGAGGAACTACCAAAATATTTTCCAAAGCAGCTGCACCTTTTTCCCATCAGCAGTGTGGGAGGTTTCAGTTTCTCCACATCCTTGCCAGCACCTGT
TATTATCTATCACAAAGTTATTTTCCAAAAACAATTCTGTGGATATAAGTGAAAATTTTAAAGGTATGGGCTTATCTTCAGTTTTTACCTCTGGCTTTTGGTTTCTGCCTTTGGAGAAAGTTGCTTTTTTCCCCGTAAAGGGCTGTGCTGTGATTTGTTTTCT
CCAGTCTCAACCCACTTGTGACCAGTGGCCCACATTCAGAAAGGGGCAAGTTTGAACCAGGAGCACTGTCCATGGCCTCGGCTTAGAGTCTTCTCTTTGTGAAGGGTCTCAAAGGAGTGGAACCTCCCCCACTGGCATAGAACTCAGAATAAGAAGCTGGCCA
GCATGTACAGTCAGTGCTGTCACTCACGGAGGTAGTCAGATATCAAACAGGAAAAGCGTGTGCCTCTTCGATTCTCCCTCTCCCTCTTGCTTTCAGATGGATGATGATGACGACGACGATGACGTAGGAGATCATGACGATGACCACCCTGGGATGGAGGTGG
TGCTGCATGAGGACAAGAAGTACTACCCAACAGCCGAGGAGGTGTATGGTCCTGAGGTGGAGACCATAGTTCAAGAGGAAGACACTCAGCCTCTCACAGGTACATCAGGAGGACATGGGCTGTGCGGAGTGAAATTGAAACCTGGAAACTTCATAGCTCTCAG
CAAACCTTAAAAGTTCAGTGAATGAATTGATCAGTTTTTCTTTACTACCTTCAGGTACTGTTTCTGGTTTAAGATTTAGTTAATCCTTTTTTAATATTCAGTAAAGTTCTTAACTAGTTTCTAAGCAAATAAGAGTTATTTCTTATTTATCTGCTATCAGTTT
AGGTGCATGAAGACTGTCGTTAAGTTTATTTACCCTGTGGCCTGATATGTTTTTCCATCCAAATAACTCCAACCCTTTTAACCAGTTCTCATAATATGTATTGTCCATTTCTTTTTCTTTTTTTGAGACGAAGTCTTGCTCTGTCGCCCATGCTGGATTACGG
TGGCACAATCTTGGCTCACTGCAACCTCCGCCTCCCTGATTCAAGCAATTCTGCTTCAGCCTCCCTAGTAGCTGGGATTATAGGTGTCTGCCACCATGCCTGGCTAATTTTTGTATTTTTAGTAGAAACGGGGTTTCACCATGTTGGCCTGGCTGGTCTTGAA
CTCCTGACCTCAAGTGATCTCTCTGCCCTGTCCTCCCAAAGAGCTGGGATTACAGGCGTGAGCCACTGCAACCAGCCTTATTGTCCATTTAATAGTTTTCATAGGCTGGAAGCGGTGGCTCATGCCTGTAATCCCAGTACTTTGGGAGGCCAGAGCAGGAGGA
TCGCTTGAACCCAGCAGTTCGAGACCAACCTGGGCAACATGGTGAAACCCCATCTCTACAAAAAAAGTACAAAAATTAGCTGGGCATGGTGGTAGATGCCTCCTGGCAACTCGGGAGACTGAGGCAGGAGGATCACTTGATCACGCCATTTGTACGCTTCAGC
CTGGGTAACAGTGAAACCCTGTCTCCCAAAAAAAAAAGTTTTCATTGATCTTTTGGAAAGTGATTGCATCTTATAAATGCAAGGAAAGATCCTGAACTGACATTGACCTCAACCTACATCCATTCCCCCTCCCTCCCTCCCACTGTCCCTCAAAATGAAGCCC
AGACTCCTCTTTCCACAATTCAAGGTCCTTCACTGTCTCATGCCAAACAGCATTTCCAAAACTTTCTCAACATATGAGTGATCCTTGAGAGTAAAATAAATTGATGTCATACATCATACATTCTGAAAGGTCAGTCTAATGGAATAGTGTTCCGTATTTTTAT
GTTTTATGCCCACCTACACATCACTACCTTTTTTTTTTCTTCTAGAACCCATTATTAAGCCAGTGAAAACCAAGAAATTCACTCTGATGGAGCAGACATTACCTGTTACGGTGTATGAGATGGAGTAAGTTACAAGGTTTCTCAGCTTCTCTGCAGGAATGCT
GTGGGTTCATGTTTTTGTAGAGATAAGAGGTGGCTTTTGGCTGAAATAGCTAGTAAATTGTGGCCGTACATAGCTCTCAAATTCTCCTTCAGCTTGTGTGGCTGCTGGGATATTTGGGGTCAATCTTAACTTTTCTCTTGGTGTTCATCCAAATGTTAGCTGT
TTCCTCTTCCAGATCTTTAGTTTGATGTTGAAAGCCAGCCTTCAAGAGAGATCTGGGTATTGCAGTTATCACTGTTGGATCCACCAGAGCCTGTTTGGACTCAATAAGACTGCTGAGTATTTTAGGAATATATTAGCTCTTTATTTTGTTTTTGTTGTTGTTA
TTTTGAGACAAGGTCTCGTTTTGTTGCTCAGGCTGTAGTGCAGTGGCACAATCATGGCTCATTGCAGCCTTGACCTCCCAGGCTCAATAGATGTGTCTGCCTCAGCCTCCTGAGCAGCTGGAACTACAGGAACACACCACCACGCCTGGCTAATTTTTGTGTT
TTTTGTACTGTTTCACTATGTTGCCCAAGCTGGTCTCAAACTCCTGGGCTCAAATGATTCTCCTGCCTTAGTTTCCCAAAGTGCTGAGATTACAGGCGTGAGCTACCATGCCCGGCCCTCAACTCTTTATTTTGAAAAGGAGAGCAAAAGGAGGTAGATATAT
GTGTAGGATCATTTAGGTGCTCTCTCTCTCTCTTTTTTTTTTTTTTCGATGCGGGGTCTCACTCTGTTGCCCAGGCTGGAGTGCAGTGGCATGATCTCAGCTCTCTACAACCTCCACCTTCCAGGTTCAAGTGATTCTCCCGCCTCAGCCTCCTGAGTAGCTGG
GATTACAGGCGCATGTCACCACGTTCAGCTAATTTTTATATTGTTAGTAGAGATGGGGTTTCACCATGTTGGAAGTCCTGGCTGGGTGTGGTGGCTCACGCTTGTAATGCCAGCACTTTGGGAGGCCAGGGCAGGTGGATCACCTGAGGTCAGGAGTTCGAGA
CCTGCCTGACCAACATGGTGAAACCCCGTCTCTGCTGAAAATGCAAAAATTAGCTGGTCGTGGTGGCCTCGGCCTCCCAAAGTGTTGGGATTACAGGCGTGAGCCTCTGCTCCCGGCCTAATTTTTGTATTTTTAGTAGAGACAGGGTTTTACCATGTTGGCC
AGGCTGGTCTTGAACTCCTGACCTCAGGTAATCCACCCACCTCGGCCTCCCAAAGTGCTGGGATCACAGGCATGAGCCACCATGCCCAGCCTCTTCTTCCTTTTTTAGTCTTGCTGTTTTACTATTGAAATCCCCGTGATAGTCCATTCACAGATTGTGTTAC
CTCTTGGATAAGTTTCTTGCATTCCCAAAGCCAGAGCTATGTAAGTATAGACATTCACATCTGCCTGAGCACTCTCCAGTTGGTCTGAAAGTTGTTCCTGGCCAAAAAATACCTGAAACACCTTTACACCACTCCTCATTTGGAAACGTTTGCCAATCTCTCT
TTTCTCTTTTCCTTTGTCCTTTCAGTTTCTTGGCGGATCTGATGGATAACTCAGAGCTCATCAGAAATGTGACCCTTTGTGGACATCTCCACCATGGCAAGGTAAGACGGAACTTTCAGCTACTTGGAACAGGATTTCTCTTTTGTACCTGCTCATGTTTTAG
CCCTTAGAGTCACAGCGGTTGAGGTTTGAGGTCACAAGGCTCAACCTCCTGACTAGGGGTGCTGCATGCCTGTTACGAAGTCATACTGTGAAATTCTGGCTTTTCCCAAGCAGCAGGTGGAGCCTGTTCCTAAGCCACTTGCATGCCTGAGCCTTATGTCTAC
ATCTGGACAGAAGATGGGAAATTGAGCCTTACTCTGATTTTTATAAGTTTTCATTTCCTGAGGGATGCCTGTCTAAGAAAGATTTCATCTTTTATTTGAAAATAATCCTGTGGCTTCAGTGTTAATTATTTGCTGTCCCATAACCCTCCTTGCTCTCCTTTTT
CTCTCCTTATCCCTAAATTATGTGGTGTATTGGGAGCTGGGGGAAGGGCACAGTAAATTTTCCTCTGTGGTCGGCAGATGTTAACTGTTTCTTCCCTTTTAGACATGTTTTGTGGATTGTTTAATTGAACAGACTCACCCGGAAATCAGAAAGCGCTATGACC
AAGATGTAAGTAGAACTTCTGTTGAGGGTTTAGGATGAACCATTCAGAACAGATAGAGATGTCTCTCTCCTCCTTCCTTCACCCTTCCTGTTCTCTATAAATAATGACGCCTCCAAAGTGCAGCTTTATAATTGTGATCTCTATTAATAGGCTGTAAGAAAAT
AATTTCAACTTTCTCGGGAGCATAGAGAATATTGAGCTGCAGTATTTTTGGATGATCCATTTTGCACTTGGGATCCCTCCTCTGTTATGATATATCCTGACTTTTATAGTCTATTCTTTCCTGTTATAGTATTAATAGCCTGCTCTATAAATATGGTACAATT
GTACCTGGTTTTCATGGTAAATTTAAATGCCTGTGAATGAGGCCAACTGTGTCTGCAAGTCTCAGTGCAATAAATGTTCAAACCCTGCCAGATTGATTGTTTTAACACTTTAGCATTAAGGCTGACATGTGTCTCCCTTTACCACACTAAGAATTCTGTGCAT
TTGATATAAATTATCTCTCAGCTATACCTGGTCTTTAGTGACCTCCCCCAACTCAAAAAGAGTGGTGGCTCATGCCTATAATCCCAGTACTTTGGGAGGCCAGGACAGGAGCATCACTTGAGGTCAGGAGTTTGAGACCAGCCTGGCCAACATGGCGAAACTC
TATCTCTACTAAAAATGCAAAATGTAGCTGGGTGGGCACGGTGGTGCACACCTGTAGTCCCAGCTACTCAAGGGGCTGAGGCAGGAGAATCGCTTGAACCTGGGTGGTAGAGGTTGCAGTGAGCCAAGATTATGCCACCACACTCTAGTCTGGGCGACTGAGT
GAGACTCCATCTCAAAAAAAGACAAAATCACAACCAAAAAGAAAAACCCAGCCATGTGTGGTGGCTCACGCCTGTAATCCCAGCACTTTGGGAGGCTGAGCCGGGGGGATCACATGAGGTCAGGAGTTCGAGACCAGCCTGACCAACATGATGAAACCCTGTG
TCTACCGAAAATACAAAATTAGCTGAGCATGGTGGCGCACGTCTGCAATCCCTGCTACTTGGGAGGCTGAGGCAGGAGAATCTCTTAAACCCTGGAGGCAAAGGAGGTTGCAGTAAGCTGAGATCGCGCCATTGCACTCCAGCCTGGGCTACAAGAGCAAAAC
TCTGTCTCCAAAAAAGTAAAAATAAAAAAATCCCTCCACACACCCAGGAGCATTTCCAGCTGGGAGGGCACTGGTCTCATTGTCTGTAATTGCTGTGGGGACACCCTGCTCTCTCTCTCTCTTTAATCTCAAAGAAACCCTTGAGCTGGGAGGTAGTGAGGGA
AAGTGATTGATGGTTCGTTTGTGGGAGAGCAGTCTCAACTCAGGTGGTAACAACAGTTAATTTTTTTCAGCTGTGCTATACTGACATCCTCTTCACAGAGCAAGAGGTAAGCATGCCTTGGGGAATCTGATTGTCACCTACACCAGACCCACCAGTAATAGT
TTTGTAGGGAATGTAGAGGAGCCTTTATGATAGCATTTTAGTTCATATCCTACTCCTCTCTCTCCACTTCTCACATCCCATCTCCTTCTTCCCCCAGTTACTCCAGGGTTATAACTTGAAAAAGAACTGGGTAGGCAAGCTGTAGGTTAGGAGGGGAACCCTC
CTGTTCTTGACTAGATTAAATTTGGCCTAATATAGGAGAAGTACTAAGAGGCGATGTCTGCATTTGTGCTATAAGTTACGCAGAAGCTCAACATGATTTTTTTAAAATGACATCATCCTTTAAAAATCCGCAGGATGAGTCCATCCATATGATGACCTCTTTC
TAGGAGTAGCTTCTCTTTGAAAATGGGTTCCTATATCTTTGTGTTTCTCTTTTTGTCTTTCCTTCCGGATCTTTCTCTCTAATCTCTGACCCCTTCTGTTTCCCTCCTCTTATTTCTGTCTCTTTTTCCTAATGGAAGTTTTAAGTATAATAAGTATGGTTAA
CTAAAGGCACTTGGAATTAGTAAAGATAGATAGGCAATCAATGTTAGCTGTTATTATTTTATAATGTATTTATGTATTTGTTTGGAGTTTTAAAAAAAAATGTTTATTTCTGTCATAGCTTTTATATTTCTTTTTTAAAAAAAAAACTAAAATACCCCAGATTGT
TTCTTTTTACTTTCAAGGTGCTCAGCTATTGTTTTTGTTTCTAATGTATAAAGAGGATGAGATTCTAGGACCCTGGATTCTCTGATAAGGCTTAATACTGTTTTGTTTGCAGTAATTCATTTTCATCTTTTCACATTTGAAACAAGTCCCCCAAAAATGGTGT
CTAACACCAAGATATTTTATCTTCAAGGTCATGCTGGCTAGAGGCCTAGATAAAAATCACCCACTATAAGGAGAGGCATCAGTGTAATTTACTAGCAACTGGGGAGCCCCAAAAACAAAATGAACCCCTCTTGCATAGGAAGAGGTCTTGACCACT

Figure S4. Sequence of genomic region of the indel in EFTUD2 in the proband. The sequence is coloured as follows: Grey = PCR primer annealing sites, Yellow = sequence in the correct orientation present in the Sanger sequencing from the proband, Green = reverse complement section 1, Blue = reverse complement section 2.

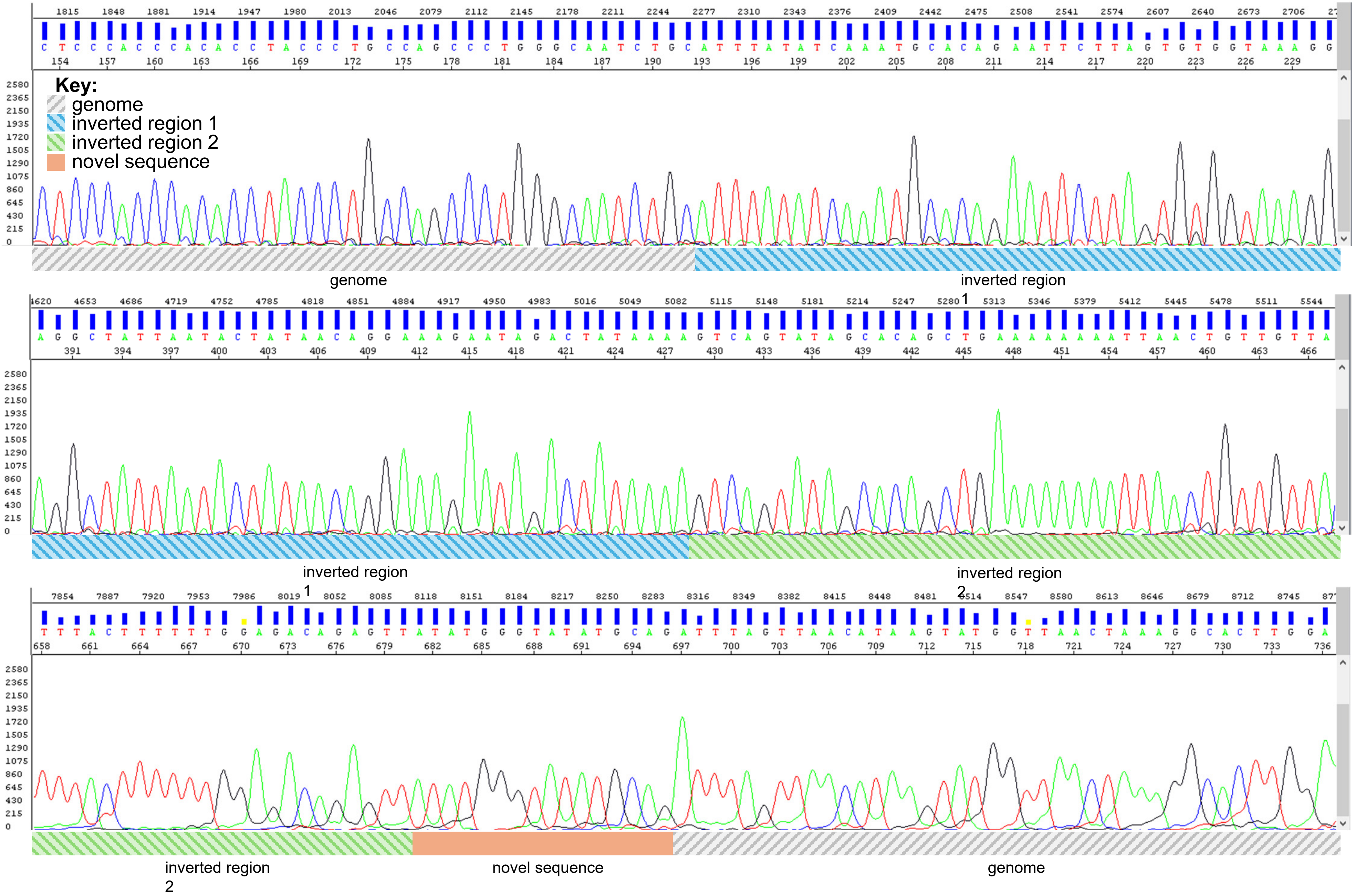

Figure S5. Sequencing chromatograms from Sanger sequencing of the PCR product shown in Fig.S2B. Selected regions are shown for clarity, see key for annotation of relevant features.
